# CRISPR-Mediated Genome Editing in Peanuts: Unlocking Trait Improvement for a Sustainable Future

**DOI:** 10.3390/plants14213302

**Published:** 2025-10-29

**Authors:** Seong Ju Han, Jia Chae, Hye Jeong Kim, Jee Hye Kim, Young-Soo Chung, Sivabalan Karthik, Jae Bok Heo

**Affiliations:** 1 Department of Molecular Genetic Engineering, Dong-A University, Busan 49315, Republic of Korea; 2 Department of Microbiology, Thiagarajar College, Madurai 625009, India; 3 National Center of Excellence in Statistical and Mathematical Modelling on Bio-Resources Management, Thiagarajar College, Madurai 625009, India

**Keywords:** genome editing, peanut species, trait advancement, CRISPR, peanut yield

## Abstract

Advancements in genome editing have transformed agricultural biotechnology by allowing for precise modifications of DNA. This technology has sparked increasing interest in enhancing important traits of major crops, including peanuts. As a nutritionally rich legume prized for its high oil content, peanut production still faces significant challenges, including disease outbreaks, nutrient deficiencies, and pest infestations. Addressing these challenges is essential for achieving high yields and sustainable cultivation. CRISPR technology, a cutting-edge genome editing tool, has emerged as a powerful platform for improving peanut traits. Its ability to facilitate gene knockouts, regulate gene expression, and introduce targeted genetic changes has accelerated research efforts in this field. The successful applications of CRISPR in peanut improvement, such as increasing oleic acid content and reducing allergenicity, reassure us about the effectiveness and potential of this technology. Despite the complexity of the peanut genome as a polyploid crop, these successes demonstrate the power of genome editing. This review emphasizes the crucial role of genome editing in enhancing peanut traits and outlines the promising future of CRISPR-based approaches in advancing peanut breeding and agricultural productivity.

## 1. Introduction

Applying advanced technologies for trait development is essential to improving global food security. A comprehensive understanding of genetic variation and the application of gene-editing techniques are critical for accelerating research and developing resilient, high-yielding crops that support both food security and sustainability [1]. Among these, genome editing represents a precision genetic technology for crop improvement, enabling the development of climate-resilient, nutritionally rich crops that address the challenges posed by global climate change [2,3].

Legumes, a major group within angiosperms, include approximately 19,500 species across 751 genera [4,5]. They are highly valued for their nutritional content, serving as an excellent source of essential amino acids and plant-based proteins. Importantly, legumes play a key role in sustainable agriculture through nitrogen fixation and soil enrichment, making them indispensable in environmentally friendly farming systems [5]. Within this family, the peanut stands out as a globally significant crop due to its high levels of iron, calcium, protein, and B-complex vitamins, including niacin, thiamine, and riboflavin [6].

Cultivated in more than 100 countries, peanut is a versatile crop valued for their oil content and wide-ranging food applications [7]. It thrives particularly well in semi-arid tropical regions [6]. The global significance of peanut production is evident in the annual harvest of over 51.3 million tonnes, with China and India leading global output (USDA, 2024). In many developing countries, peanuts play a crucial role in combating poverty and malnutrition by providing essential proteins, calories, vitamins, and minerals [7,8]. It is often cultivated on marginal soils, with limited resources, and intercropped with cereals [6].

Recent research has proposed peanut-based formulations as ready-to-use therapeutic foods (RUTFs) to combat protein-energy malnutrition in children [9]. With a low glycemic index of 14, peanuts are also recognized for their potential to reduce the risk of diabetes and cardiovascular disease [10]. Furthermore, peanuts contain various bioactive compounds, including stilbenes, phytosterols, lignans, and isoflavonoids, which provide health benefits and disease prevention properties [11]. Due to these attributes, peanuts are increasingly regarded as a functional food [12]. Resveratrol, a prominent stilbene found in peanuts, offers antioxidant, anticancer, and anti-inflammatory properties, contributing to cardiovascular health [12]. Additionally, peanut oil is rich in oleic acid, a monounsaturated fatty acid that enhances cardiovascular health and extends shelf life compared to polyunsaturated linoleic acid. The versatility and nutritional value of peanuts have led to their widespread use in multiple forms, including raw, roasted, salted, peanut butter, confections, and snack products [9]. Traditional breeding efforts have improved peanut productivity, quality, and resistance to biotic and abiotic stresses [7]. However, genetic improvement in peanuts has not progressed as rapidly as in other crops. To overcome this lag, there is an urgent need to accelerate research using advanced tools, particularly genome-editing technologies.

### 1.1. Genome Editing Tools

Various genome editing tools have been developed to introduce targeted genetic variations in crop genomes. These tools include meganucleases (MNs), zinc finger nucleases (ZFNs), transcription activator-like effector nucleases (TALENs), and clustered regularly interspaced short palindromic repeats (CRISPR). Each of these tools holds great promise for enhancing agricultural productivity and ensuring global food security. MNs stand out as the pioneering tool in this field, capable of recognizing long DNA sequences (12 to 40 bp) with impressive specificity. However, adapting MNs for new targets can be a challenging task. In contrast, the flexibility of ZFNs and TALENs is notable. ZFNs have been used to edit the *AHAS* gene in wheat, providing resistance to imazamox [13]. ZFNs edited the *ALS, SuRA,* and *SuRB* genes in tobacco, conferring resistance to imidazolinone and sulphonylurea herbicides [14]. TALENs have been applied in wheat to knock out the *TaMLO* gene, conferring resistance to powdery mildew [15]. Targeted editing of the *ALS1* gene, using sequence-specific nucleases delivered via a geminivirus replicon, conferred herbicide tolerance in potatoes [16]. TALENs targeted caffeic acid *O-methyltransferase* to reduce lignin and improve biofuel production in sugarcane [17]. The *OsBADH2* gene was edited to enhance aroma in rice [18]. Despite their effectiveness, traditional genome editing methods, such as ZFNs and TALENs, are limited by their complexity, high costs, and lower editing efficiency. Conversely, the CRISPR/Cas system has emerged as a more versatile, efficient, and user-friendly platform that enables precise and cost-effective genome editing. The maize gene *ARGOS8* has been edited to enhance drought tolerance [19]. Similarly, disrupting the *SlMAPK3* gene in tomatoes reduced drought tolerance [20] and editing the *NOR* gene in tomatoes delays ripening and softening [21]. In rice, knocking out the *OsNAC041* transcription factor with CRISPR/Cas9 increased salt sensitivity, demonstrating its role in salt stress responses [22]. Knocking out genes such as *SGR1*, *LCY-E*, *BLC*, *LCY-B1*, and *LCYB2* has increased lycopene content by over fivefold in tomato [23]. Editing the *DEP1* gene in rice enhances yield by affecting panicle size [24]. CRISPR/Cas9 knockout of *LOX* genes in barley boosted grain fatty acid content and storability, demonstrating the potential of genome editing to enhance energy-rich seed traits in cereals [25]. CRISPR/Cas9 editing of *ahFAD2A/2B* in peanuts effectively disrupted oleate desaturation, leading to increased oleic acid levels (approximately 85–90%) and enhanced oil stability, as demonstrated by Neelakandan et al. (2022) [26]. These findings are further supported by additional studies reporting similar high-oleate phenotypes and improved oil quality through *FAD2*-targeted editing [27,28,29,30] (Table 1). Previous reports suggest that CRISPR/Cas is a straightforward and effective tool for enhancing peanut traits and promoting sustainable agriculture.

### 1.2. Class of CRISPR/Cas Systems

CRISPR/Cas systems are categorized into two main classes, comprising six types and several subtypes, based on their genomic architecture and the unique Cas genes present within them. Class I includes Types I, III, and IV, which utilize multiple Cas subunits for nucleic acid interference. In contrast, Class II encompasses Types II, V, and VI, which rely on a single, highly efficient effector complex combined with guide RNAs (gRNAs). Among the various CRISPR systems, the Type II system is undeniably the most prominent and widely adopted. It was discovered in the bacterium Streptococcus pyogenes, and features a single guide RNA (sgRNA) that is a synthetic fusion of CRISPR RNA (crRNA) and trans-activating CRISPR RNA (tracrRNA) [39]. It works in tandem with the Cas9 protein to execute precise cuts in specific DNA locations [39]. A key component in this operation is the protospacer adjacent motif (PAM), which allows Cas9 to recognize its target effectively [39]. The enzyme expertly identifies PAM sequences, unwinding the DNA to make exact cuts. Specifically, Cas9 targets the NGG nucleotide sequence as PAM, activating its unique catalytic domains (HNH and RuvC) to generate double-stranded breaks in the genetic material of unwanted elements [40]. These precise double-stranded breaks are a valuable tool in genomic research, enabling scientists to create targeted point mutations linked to specific traits. The CRISPR/Cas9 process consists of two main steps. First, the Cas9 enzyme, guided by a small RNA molecule, creates double-stranded breaks at specific locations in the DNA [40]. Second, the cell’s natural DNA repair mechanisms are activated [40]. One mechanism, known as non-homologous end joining (NHEJ), is often error-prone and can result in small insertions or deletions (indels) at the break site, potentially disabling or altering the targeted gene. Alternatively, the more accurate homology-directed repair (HDR) pathway repairs the break in a way that allows for gene insertion or replacement [39]. HDR is crucial for successful CRISPR-based mutations, as it enables the precise incorporation of donor DNA to correct the targeted site [40]. Additionally, researchers are exploring the promising Type III Cas10 variant, characterized by its HD family nuclease domain. This variant differs from those found in Type I CRISPR-Cas systems and features a unique circular arrangement of conserved motifs [41].

### 1.3. Innovations on Cas Variants

CRISPR/Cas12 has also made significant strides in gene editing for crops, starting with its application in *Clostridium difficile*, which enables efficient multiple genome modifications [40]. The Cas13 variant is particularly notable for using guide RNA that has dual catalytic functions: RNase activity and gRNA maturation. Its compact size opens up numerous possibilities for various applications in molecular genetics [42]. Cas13 is recognized for targeting specific RNase activity and its ability to cleave single-stranded RNA, similar to the functions observed in types II and V CRISPR systems. It has been incorporated into plant genome-editing research [43]. Cas12a/Cpf1 systems from *Lachnospiraceae bacterium* (LbCas12a), *Acidaminococcus* sp. BV3L6 (AsCas12a) and *Francisella tularensis* novicida (FnCas12a) are commonly used for genome editing in various plant species [44]. These systems demonstrate high success rates, boosting confidence in their effectiveness. Consistently, CRISPR/Cpf1-mediated genome editing has shown significant promise in both mammalian cells and plants, utilizing various Cpf1 variants. This technology has successfully achieved multiplexing through the use of a crRNA array with both Pol II and Pol III promoters [45]. Notably, crRNA production from Pol II promoters can reach editing efficiencies that are equal to or even greater than those from Pol III promoters. This is likely due to more efficient export of Pol II transcripts to the cytoplasm, which enhances interactions between crRNA and Cpf1 [46]. Using multiple U3 or U6 promoters in gRNA expression cassettes in plants can lead to variability in gRNA expression and transgene silencing [47]. The individual gRNA cassette method is also constrained by plasmid cloning efficiency and insert size, particularly with viral delivery systems [48]. While binary vectors for mediated transformation have fewer restrictions on transgene size, the random insertion of multiple gRNA cassettes at a single locus can lead to transgene silencing [49]. Although multiple DNA plasmids can deliver gRNA expression cassettes, this approach often results in low efficiency and cytotoxicity [50,51]. Most *Agrobacterium* strains possess a single T-DNA plasmid, which complicates the delivery of multiple plasmids to different organisms [52]. Thus, creating a compact plasmid that expresses various gRNAs or crRNAs is preferable. Furthermore, the CRISPR/Cas ribonucleoprotein (RNP) complex enables genome editing without the use of plasmids, allowing for precise control and preventing the integration of foreign DNA [51]. This method involves delivering purified Cas9 nuclease along with either in vitro transcribed RNA or chemically synthesized single guide RNAs (sgRNAs), into target cells via direct injection or synthetic nanoparticles [53]. PEG-mediated transfection has successfully introduced RNPs into *Arabidopsis* and rice protoplasts [54], although it has limitations with plants that have low protoplast regeneration. Genome editing has been successfully achieved in wheat and maize through the co-bombardment of RNPs along with transcription factors [55]. Recent research indicates that nanomaterials, such as carbon dots (approximately 10 nm) and carbon nanotubes, can effectively deliver plasmids and RNPs into mature plant cells. This delivery can be achieved through methods such as spraying, leaf dipping, or infiltration [56].

Research indicates that Cas9 is the preferred tool for developing genome-edited plants, with its expression under the cauliflower mosaic virus (CaMV) 35S promoter referenced in 78 studies on plant genome editing [57,58]. Qi et al. (2013) introduced mutations in two endonuclease domains of the Cas9 protein within the CRISPR/Cas9 system, creating a version known as dead Cas9 (dCas9) [59]. They achieved this by substituting aspartic acid at position 10 of the RuvC domain (D10A) and changing histidine at position 840 of the HNH domain to alanine. Although dCas9 cannot cleave DNA, it can still bind to specific target sequences guided by RNA [58]. When dCas9 binds to the promoter transcription start site (TSS), it blocks RNA polymerase and transcription factors from accessing the promoter, thereby inhibiting gene expression without altering the genome. This process is referred to as CRISPR interference (CRISPRi) [60]. The level of inhibition can be as high as 1000-fold, primarily in prokaryotes, and this technique has also been applied in plants such as *Nicotiana tabacum, Zea mays, and Arabidopsis thaliana* [61,62,63]. Transcriptional activation, also known as CRISPRa or CRISPR activation, can be accomplished using dCas9. Furthermore, Bikard et al. (2013) showed that fusing dCas9 with the ω subunit (*rpoZ*) in *E. coli* increased the transcription levels of a reporter gene by as much as 2.8-fold [63,64].

Over the past decade, the field of CRISPR-based plant genome editing has made significant progress. This is an ideal time to reflect on these developments and highlight recent innovations that enhance the efficiency of genome editing in plants. Advancements in genome editing are revolutionizing traditional breeding processes, enabling us to enhance peanuts for various traits. This review highlights promising advances in genome editing aimed at improving peanut traits.

## 2. Taxonomy of Peanut

*Arachis hypogaea* L., commonly known as peanut, is an annual legume belonging to the family Fabaceae (Leguminosae). It comprises two subspecies: *A. hypogaea* subsp. *fastigiata* Waldron and subsp. *hypogaea* Krap. et. Rig. The Subsp. *fastigiata* is further divided into four botanical varieties (*peruviana*, *vulgaris*, *fastigiata*, and *aequatoriana*), while subsp. *hypogaea* includes *hirsuta* and *hypogaea* [7,9]. Krapovickas and Gregory (1994) [65] described diverse morphological traits in plants, pods, and seeds across these categories.

Peanut is an allotetraploid species (2n = 4x = 40) with an ‘AA’ and ‘BB’ genome constitution [7]. Among the Arachis section, only *A. hypogaea* and its wild relative, *A. monticola*, are tetraploids; all other species are diploids. The diploid ancestors *A. duranensis* (AA genome) and *A. ipaensis* (BB genome) are believed to have contributed to the origin of cultivated peanut [66,67]. Molecular evidence from intron sequences and microsatellite markers supports the hypothesis that a hybridization event between these progenitors approximately 3500 years ago gave rise to the tetraploid genome of the cultivated peanut [68]. The center of origin of *A. hypogaea* is proposed to be southern Bolivia and northern Argentina. In contrast, the broader center of diversity for the genus encompasses western Brazil, Bolivia, Paraguay, and northern Argentina [69]. *A. duranensis* is widely distributed in this region, whereas *A. ipaensis* is endemic to southern Bolivia [7]. Singh and Simpson (1994) [70] categorized the *Arachis* genus into four gene pools: (1) the primary pool includes *A. hypogaea* and *A. monticola*; (2) the secondary pool comprises diploid *Arachis* species that are cross-compatible with *A. hypogaea*; (3) the tertiary pool consists of procumbent species with limited compatibility; and (4) the quaternary pool encompasses wild *Arachis* species from other taxonomic sections.

Peanut is predominantly self-pollinated, producing cleistogamous flowers. However, occasional natural hybridization can occur via insect pollinators such as bees [71]. Depending on genotype and environmental conditions, flowering typically begins 17–35 days after seedling emergence. Flowers arise only in leaf axils and not on vegetative branches, with one or more flowers per node. The stigma becomes receptive approximately 24 h before anthesis and remains receptive for 12 h after. Anther dehiscence occurs 2–3 h before flower opening at dawn. Fertilization begins roughly six hours post-pollination. The developing gynophore, carrying the fertilized ovule, elongates and grows geotropically into the soil within 6–10 days, depending on temperature. Optimal peanut growth requires well-distributed rainfall of at least 500 mm and an air temperature of 25–30 °C. Since pod development occurs underground, calcium is essential for proper kernel formation. To address this, calcium is typically applied directly to the pod zone during peak flowering, where it is rapidly absorbed by developing pods [7].

## 3. Genome Structure of Peanut

Extensive research has revealed that the genome size of *Arachis hypogaea* is approximately 2.7 Gb. This size is nearly equivalent to the combined sizes of *A. duranensis* (1.25 Gb) and *A. ipaensis* (1.56 Gb), suggesting minimal changes in genome size after polyploidization. The homologous chromosomes of *A. duranensis* and *A. ipaensis* species exhibit a strong one-to-one correspondence, with 02/12, 03/13, 04/14, and 10/20 demonstrating near-perfect collinearity [72]. In contrast, chromosomes 06/16 and 09/19 show prominent inversions in one of their arms. Two large inversions characterize chromosomes 05/15, while chromosomes 01/11 contain three major inversions. Additionally, chromosomes 17 and 18 underwent reciprocal translocations near chromosomes 07/08 [73]. The distal ends of chromosomes are gene-rich, with the B subgenome harboring approximately 11% more genes than the A subgenome. Precisely, the B subgenome has 35,110 predicted genes, compared to 31,359 in the A subgenome. Long-term repeat retrotransposons are abundant in pericentromeric regions, while DNA transposons are more prevalent in euchromatic arms [72]. *A. duranensis* has contributed both the chloroplast genome and a chloroplastic plasmid to *A. hypogaea*. Gene methylation patterns are typical of those observed in other plants, characterized by reduced methylation in transcribed regions and significant decreases at transcription start and stop sites. Pericentromeric regions exhibit higher cytosine methylation levels than chromosomal arms. Moreover, the A subgenome displays lower methylation than the B subgenome. At CG sites, methylation levels are 76.0% in the A subgenome and 80.5% in the B subgenome. At CHG sites, levels are 61.7% and 65.1%, respectively, while at CHH sites (where H represents A, T, or C), they are 5.14% and 5.51%. Additionally, many short RNAs are enriched in repetitive regions, whereas unique small RNAs are found in the gene-dense areas of the chromosomes [9,72].

## 4. Improvement of Peanut Traits

Genetic studies have identified key loci associated with yield in peanuts, while advanced sequencing technologies have revealed significant sequence variations in genes related to these traits. The discovery of advantageous alleles provides exciting opportunities to use genome engineering to enhance these traits. This breakthrough in genome editing not only improves peanut characteristics but also has the potential to significantly boost food security, offering a hopeful perspective for the future. The following sections will discuss how genome editing could improve peanut traits.

### 4.1. Reduction of Aflatoxin Contamination in Peanut Seeds

The susceptibility of peanut crops to aflatoxin contamination caused by *Aspergillus flavus* infection has been extensively studied. Effective management of both pre- and post-harvest conditions can prevent seed invasion and subsequent aflatoxin accumulation, although various environmental stresses tend to exacerbate the issue. Understanding the genetic basis of aflatoxin resistance remains a significant challenge and focus, as it is critical to the development of more resistant cultivars. In one study, researchers successfully employed RNA interference (RNAi) to suppress five key genes involved in aflatoxin biosynthesis (*aflR, aflS, aflC, pes1,* and *aflep*), resulting in a complete (100%) reduction in aflatoxin B1 and B2 production [74]. Additional efforts have included the overexpression of antifungal defensins *MsDef1* and *MtDef4.2* to enhance aflatoxin resistance in peanut [74]. Host-induced gene silencing (HIGS) targeting *aflM* and *aflP* has also been used to inhibit aflatoxin production post-infection [75]. Another study applied HIGS to silence genes involved in fungal morphogenesis and aflatoxin biosynthesis, resulting in plants with significantly reduced infection levels and aflatoxin contamination (less than 20 ppb) [76]. However, these RNAi and HIGS-based strategies still face significant challenges, including incomplete silencing and inconsistent performance in field conditions, underscoring the need for more effective solutions. The *AhAftr1* (*Arachis hypogaea Aflatoxin Resistance* 1) gene has recently been identified as a key factor in activating disease resistance through an immune signaling pathway [31]. Moreover, the potential of the CRISPR system in reducing aflatoxin contamination in peanut seeds is a promising area of research. This finding highlights potential targets for accurate genome editing to minimize aflatoxin contamination in peanut seeds. Hence, the CRISPR/Cas system serves as a powerful platform for directly manipulating both host resistance genes and pathways involved in fungal interactions, facilitating the development of durable, aflatoxin-resistant peanut cultivars.

### 4.2. Reduction of Allergen Genes in Peanut Seeds

Food allergies can lead to severe reactions and impose significant healthcare costs [77]. In the United States, an estimated 3–8% of children are affected by food allergies, with prevalence on the rise [78,79]. While early introduction of allergenic foods may help prevent the development of allergies, strict avoidance remains essential for individuals who are already sensitized [79,80]. Among food allergies, peanut allergy is particularly concerning due to its potential to trigger life-threatening anaphylactic reactions [81]. Approximately 2% of children in the U.S. have a peanut allergy, and its prevalence appears to be increasing [81,82]. Unlike allergies to cow’s milk or hen’s egg, which often resolve by adolescence, peanut allergy frequently persists into adulthood. For sensitized individuals, strict avoidance of peanuts or the use of allergen immunotherapies such as oral immunotherapy or FDA-approved treatments like Palforzia are recommended management strategies [78].

Recent advancements in molecular biotechnology have opened promising avenues for reducing peanut allergenicity. One such approach involves RNA interference (RNAi), a gene-silencing technique that reduces gene expression at the level of mRNA. Researchers have utilized RNAi to target *Ara h 2*, a major allergenic glycoprotein in peanuts [82,83]. Using *Agrobacterium*-mediated transformation, an RNAi-expressing plasmid was introduced into peanut plants, achieving stable integration in 44% of the transgenic lines [83,84]. Seeds from these modified plants contained approximately 25% less *Ara h 2* protein compared to wild-type controls, resulting in significantly reduced binding of IgE antibodies from peanut-allergic patients [78,83]. Recently, Conner et al. (2024) successfully utilized CRISPR/Cas9 for the multiplex editing of *Ara h 2, Ara h 6*, and *Ara h 7* (Table 1) in peanut seeds, without adversely affecting plant growth [32]. This achievement marks a significant milestone in peanut allergy research, offering both validation of genome editing tools in legume crops and optimism for future therapeutic and breeding strategies. Future research suggests that utilizing CRISPR-based genome editing with reduced immunogenicity in low-allergen genotypes may help preserve essential traits, such as flavor, nutrition, and productivity.

### 4.3. Seed Dormancy

Understanding the role of seed dormancy is essential for improving peanut production [9]. Dormancy acts as a natural defense mechanism that prevents premature seed germination before harvest. In Spanish-type peanut genotypes, the absence of dormancy increases susceptibility to pre-harvest sprouting under wet conditions, ultimately reducing both yield and seed quality. Dormancy is particularly beneficial during unexpected rainy periods, as it allows for delayed harvesting, minimizing potential losses and helping to secure better market prices. Breeding cultivars with controlled or reduced dormancy durations offers a sustainable and economically viable strategy to address these challenges in peanut cultivation. Recent studies have identified two genomic regions on chromosomes A09 and B05 associated with fresh seed dormancy (*FSD*) in inbred lines derived from a biparental cross [85]. Two candidate gene proteins, finger protein (*RING-H2*) and zeaxanthin epoxidase (*ZEP*), were identified as playing key roles in *FSD* regulation. Furthermore, a molecular marker, *GMFSD1*, was successfully developed to aid in the selection of dormancy traits [85].

Using the Axiom Arachis 58K SNP array, a recombinant inbred line (RIL) population analysis revealed two significant quantitative trait loci (QTLs) for FSD on chromosomes *A04* and *A05*, accounting for 43.16% and 51.61% of the phenotypic variance, respectively [86]. Additionally, a recent study utilizing a 5K mid-density genotyping assay on an RIL population derived from the cross between ICGV 02266 (non-dormant) and ICGV 97045 (dormant) identified five major QTLs associated with seed dormancy on chromosomes *Ah01*, *Ah06*, *Ah11*, *Ah16*, and *Ah17* [87]. The identification of these QTLs provides a framework for understanding seed dormancy in peanut plants. Notably, loci found on chromosomes *A09* and *B05* contain candidate genes, including the *RING-H2* and *ZEP*. These genes play essential roles in hormonal pathways involving abscisic acid (ABA), which is a key factor in regulating dormancy and germination.

Major-effect QTLs located on chromosomes *A04*, *A05*, *Ah01*, *Ah06*, *Ah11*, *Ah16*, and *Ah17* account for over 40% of the phenotypic variance, making them prime targets for further research. These genomic regions likely contain regulatory elements that influence the balance between dormancy and germination. Concentrating on these specific genetic loci for CRISPR-based gene editing could enable customized adjustments to dormancy duration, thereby improving the development of peanut varieties that can thrive in diverse conditions. For instance, modifying the *ZEP* gene could enhance *ABA* metabolism, reducing pre-harvest sprouting while preserving seed viability. Combining QTL data with CRISPR techniques will enable the development of peanut cultivars with controlled dormancy, enhancing both harvest flexibility and yield stability.

### 4.4. Low Phytate Content

Peanut kernels are rich in essential micronutrients, such as iron (Fe) and zinc (Zn); however, their bioavailability is significantly hindered by high levels of phytic acid (PA) [88,89]. Phytic acid plays a crucial role in storing phosphorus (P), which is essential for seed development [88]. Therefore, strategies to reduce PA content without negatively impacting plant metabolism or yield are of great importance. PA is primarily synthesized via both lipid-dependent and lipid-independent pathways. In legumes, seed PA is mainly produced through lipid-independent pathways [90]. When applying CRISPR/Cas9-mediated targeted mutagenesis to modify the PA biosynthetic pathway, careful selection of the target gene is critical. Early-pathway enzymes such as *myo*-inositol phosphate synthase (MIPS) and *myo*-inositol kinase (MIK) are essential for *myo*-inositol production, and their disruption can result in severe pleiotropic defects, as observed in soybeans [89]. Additionally, MRP genes regulate the compartmentalization of PA, and mutations in these genes can lead to metabolic imbalances in soybeans [9,56]. Among the downstream enzymes, *inositol pentakisphosphate 2-kinase 1* (IPK1) catalyzes the final step in PA biosynthesis (conversion of IP5 to IP6) and is a key target for developing low-phytate mutants [91]. Another critical enzyme, *inositol tetrakisphosphate kinase* (ITPK), mediates the penultimate step in the PA biosynthetic pathway (IP3 to IP5). Successful gene editing of these enzymes has been demonstrated in soybean and oilseed rape, representing significant advances in the field [92,93]. CRISPR/Cas9-based targeted peanut mutagenesis presents a promising strategy to enhance global micronutrient bioavailability by addressing the above challenges associated with PA accumulation, thereby contributing to the worldwide effort to combat hidden hunger.

### 4.5. Improve Oleate Content

The enzyme *FAD2* (fatty acid desaturase) catalyzes the conversion of oleic acid to linoleic acid, playing a crucial role in the insertion of a second double bond [94]. In the A and B genomes, this enzyme is encoded by two homologous genes, *ahFAD2A* and *ahFAD2B* [95]. Reducing *FAD2* activity, which increases the oleic to linoleic acid (O/L) ratio, can enhance the oleic acid content in peanut oil [9], thereby improving oil quality and offering significant health benefits. However, conventional breeding methods using mutagens may induce unintended mutations across the genome. The CRISPR/Cas9 system, known for its precision and efficiency, is a powerful tool for generating novel *FAD2* mutants in peanut, enabling the development of high-oleate lines. The system’s accuracy and reliability position it as a transformative technology for targeted genetic modification. The *ahFAD2* genes in peanut have been extensively studied. Yuan et al. (2019) [33] reported on the application of mutagenesis in the peanut genome to develop high oleate lines. The study focused on gene hotspots that are commonly influenced by natural mutations, using CRISPR/Cas9-based gene editing to create three specific mutations, including G448A and an insertion of “A” between positions 441 and 442 (denoted as 441_442insA). Existing high-oleate peanut varieties align with these mutations [95]. Two specific types of mutations were identified in the *ahFAD2A* and *ahFAD2B* genes. The first mutation is a substitution of “G” to “A” occurring 448 base pairs after the start codon in the *ahFAD2A* gene (G448A). The second mutation is an insertion of “A” between positions 441 and 442 (441_442insA) in the *ahFAD2B* gene [33]. The 441_442insA mutation, along with a new mutation G451T induced by CRISPR/Cas9 gene editing in the coding region of *ahFAD2B*, may be beneficial for breeding programs focused on enhancing the high oleate trait in peanuts [95]. This is particularly significant because the 441_442insA mutation has already been characterized and accepted. The phenotypic effects of these mutations must be confirmed in fully regenerated plants. Once validated, CRISPR/Cas9-induced mutations in the *ahFAD2B* gene may help increase oleic acid content in peanuts, especially if the plant line also has the pre-existing G448A mutation in the *ahFAD2A* gene [95]. Another promising strategy to elevate oleic acid levels involves targeting the *AhFatB* genes using CRISPR/Cas9. Mutations in *Arahy. 4E7QKU* has been associated with reduced palmitic acid and increased oleic acid content [27]. In a recent study, node injection of CRISPR/Cas9 constructs targeting *FAD2B* resulted in the production of two F_1_ seeds with an oleic acid content exceeding 80%. This was achieved via insertion of 442A, resulting in loss-of-function mutations in *FAD2B* [30]. Furthermore, identifying key genes associated with oleate content enables the use of CRISPR to enhance oil production in peanuts.

### 4.6. Productivity

Flowering management plays a critical role in determining peanut seed yield. This understanding not only deepens our knowledge of plant biology but also holds significant potential for practical applications in crop improvement. Many plants rely on specific environmental cues such as temperature and photoperiod (the length of day and night) to initiate flowering [96]. Photoperiodic flowering, in particular, allows plants to synchronize flowering with seasonal changes, which is essential for successful reproduction and adaptation [96]. Peanut plants exhibit rapid growth, typically reaching maturity within 40 to 100 days. During this period, their foliage expands four to fivefold. Flowering generally begins 25 to 40 days after planting. Uniquely, peanuts develop their pods and seeds underground, originating from the base of the flower.

In *Arabidopsis thaliana*, extensive research has uncovered several genetic pathways that regulate floral signaling [97,98]. Various flowering-related genes respond differently to environmental and endogenous signals [3]. Andrés and Coupland (2012) [97] identified a floral integrator pathway involving genes such as *TWIN SISTER OF FT* (*TSF*), *SUPPRESSOR OF OVEREXPRESSION OF CONSTANS1* (*SOC1*), *FLOWERING LOCUS T* (*FT*), and *OVEREXPRESSION OF CONSTANS1 (SOC1*). In both long-day (LD) and short-day (SD) plants, the *FT* gene and its ortholog *Hd3a* act as central integrators of multiple flowering signals [3,98]. *FT* orthologs have been discovered in peanuts and other plant species, including pea, kiwi, tomato, rose, strawberry, and poplar, underscoring their conserved and critical role in the flowering process. A recent study by Kang et al. (2019) utilized orthologous gene analysis to identify several key flowering regulators in peanuts, including *GIGANTEA* (*GI*), *CONSTANS* (*CO*), and *EARLY FLOWERING 4* (*ELF4*) [99]. These findings significantly advance our understanding of the genetic networks governing flowering time in peanuts. Furthermore, the identification of these genes opens new possibilities for targeted genome editing using CRISPR technology, offering a promising avenue for enhancing peanut productivity and supporting future breeding efforts.

### 4.7. Biotic and Abiotic Stress Tolerance

Peanuts face significant threats from various biotic stresses, including fungal, bacterial, and viral pathogens, as well as insect pests. These challenges result in a decrease in both yield and quality [100]. To combat these issues, researchers have introduced earlier reports on cry genes such as *Cry1AcF*, *Cry1EC*, and *Cry1X* into the peanut genome to enhance resistance against pests like *Spodoptera litura* and *Helicoverpa armigera* [101,102,103,104]. The combination of *Cry1EC* and *Chi11* also targets *Phaeoisariopsis personata* and *Spodoptera litura*, while *Cry8Ea1*, along with the *MARs*, is effective against *Holotrichia parallela* [105,106,107]. Transgenic peanuts that express several proteins, including *SniOLP* (*Solanum nigrum osmotin-like protein*), *Rs-AFP2* (*Raphanus sativus antifungal protein2*), and various β-1,3-glucanases from Arabidopsis, alfalfa, and tobacco, as well as *AdSGT1* from *Arachis diogoi*, have shown resistance to the fungus *Phaeoisariopsis personata* [108,109,110,111,112]. Moreover, peanuts expressing the antisense *nucleocapsid (N)* gene from the *Tomato Spotted Wilt Virus* (TSWV) demonstrate resistance to TSWV, while those expressing the *PBNV-N nucleocapsid* gene exhibit resistance to *Peanut Bud Necrosis Virus* (PBNV) [113,114].

Fungal diseases, such as early leaf spot (ELS), late leaf spot (LLS), and web blotch, have a significant impact on peanut cultivation worldwide. QTLs have been identified, providing insights into genetic resistance: the stable QTL, qLLS.LG02q, on chromosome 2, enhances resistance to LLS [115], while qLLSB03 and qLLSB05 on chromosomes A03 and B05 confer resistance to both ELS and LLS [116]. Studies suggest that the genetic mechanisms for these diseases are mainly independent [116]. For web blotch, major QTLs qWBRA04q and qWBRA14q are associated with NBS-LRR-type resistance genes [117]. In terms of viral and bacterial pathogens, the *PSWDR-1* locus on chromosome A01 confers resistance to TSWV and contains several promising candidate genes, including an NBS-LRR gene [118]. The identification and fine mapping of these QTLs open new avenues for using CRISPR/Cas9 genome editing to enhance disease and pest resistance in peanuts. The *MLO-like* gene (*Arahy.FX71XI*) related to TSWV resistance is particularly notable, as disrupting *MLO* genes has led to durable resistance in peanut [118]. Furthermore, targeted editing of *NBS-LRR* genes can strengthen or broaden resistance against diverse pathogens. Therefore, combining QTL mapping with genome editing can accelerate the development of peanut varieties with robust and broad-spectrum disease resistance.

Environmental factors, such as temperature and water availability, are crucial for the successful growth and development of peanuts. Ensuring optimal conditions in these areas can significantly enhance peanut yields and quality. Among these, particularly heat and drought, have a profound impact, directly limiting crop yield [119]. Peanuts are predominantly cultivated in warmer climates, where the combination of heat and drought stress poses a major challenge, ultimately leading to significant yield reductions [9]. Peanut is also moderately sensitive to salinity, with a narrow tolerance range. Soil electrical conductivity (ECe) levels exceeding 4 dS/m are detrimental to plant health and productivity, underscoring the urgent need to enhance the crop’s resilience to abiotic stress [119].

Recent advances in genetic transformation techniques have opened promising avenues for improving stress tolerance. A notable study demonstrated the effectiveness of combining pollen tube pathway transformation with *Agrobacterium tumefaciens*-mediated gene transfer. This approach was employed to deliver CRISPR/Cas9 constructs targeting *AhMULE9A*, a mutator-like transposable element implicated in the plant’s response to aluminum stress [34]. Another study identified the transcription factor *AtDREB* as a key regulator of drought and salinity tolerance. *AtDREBI* enhances stress resilience by upregulating genes involved in reactive oxygen species (ROS) scavenging and reducing lipid peroxidation, thereby mitigating cellular damage [35].

CRISPR/Cas9 is more than just a gene-editing tool; it is a transformative platform for peanut improvement. Its application offers unprecedented opportunities to develop stress-resilient cultivars adapted to changing environmental conditions. However, realizing its full potential requires the systematic identification and functional characterization of genes involved in abiotic stress response pathways. This foundational step will pave the way for the next generation of climate-smart peanut varieties.

## 5. Applications of Genome Editing in Enhancing Peanut Traits

The world of agriculture is continuously evolving, and it is exciting to see how genome editing is dramatically transforming crops. While many crops have benefited from these advancements, peanuts have seen relatively fewer reports of improvement until recently. In recent years, significant progress in genome editing techniques specific to peanuts has opened up a world of possibilities.

Traditionally, methods such as ZFNs and MNs have encountered challenges in modifying peanuts, primarily due to the complexities of designing and constructing vectors. However, TALENs have made impressive strides by targeting the fatty acid desaturase 2 (*ahFAD2*) gene. This gene plays a vital role in converting monounsaturated oleic acid into polyunsaturated linoleic acid. Increasing oleic acid levels has become a primary focus in peanut breeding due to its health benefits, including antioxidant properties and cholesterol-lowering effects, which are appealing to both consumers and industries. Additionally, peanuts with higher oleic acid content promise enhanced flavor, improved nutritional quality, and a longer shelf life [120]. Until now, most high-oleic acid peanut genotypes have been derived from natural genetic mutations in the *ahFAD2* gene. The gene-editing approach opens new avenues to induce additional mutations in this gene. A notable study by Wen et al. (2018) demonstrated the potential of TALENs, resulting in mutant peanut lines with oleic acid increases ranging from 42.5% to an impressive 92.5% [36].

Looking ahead, research by Yuan et al. (2019) utilized CRISPR/Cas9 technology in peanut protoplasts and hairy root cultures, resulting in exciting mutations in the *AhFAD2* gene [33]. Shu et al. (2020) further validated crucial nodulation genes, *AhNFR1* and *AhNFR5*, using the same sophisticated CRISPR/Cas9 method, proving the system’s versatility and effectiveness [37]. The ability to make precise modifications through techniques like base editing or prime editing is particularly noteworthy. These advanced techniques enable the accurate introduction of point mutations without inducing harmful double-stranded DNA breaks (DSBs) [121]. For instance, Neelakandan et al. (2022) demonstrated the use of a cytosine base editor to target *cis*-regulatory elements and coding regions in the *AhFADH2* gene, showcasing the potential for tailored improvements in peanuts [38]. Additionally, Neelakandan et al. (2022) [26] achieved site-specific genome modification of the *AhFAD2* gene, resulting in T_0_ seeds with oleic acid content ranging from 55% to 70%. However, it is essential to note that the transformations observed in T_0_ seeds exhibited instability in the T_1_ generation, indicating that further refinements are necessary for germline inheritance [26].

Furthermore, prime editing has been successfully applied in peanuts to restore the *GFP* gene [28]. It is essential to recognize that stable transgenic plants have yet to be produced in the studies mentioned above, as much of the research has focused on protoplasts, hairy root systems, or injection methods. However, two notable studies have successfully generated stable transformants in peanuts using CRISPR/Cas9 technology. One of these studies reported a remarkable 80% increase in oleic acid content in T_1_ seeds from plants where the *AhFAD2B* gene was knocked out [30]. Another innovative project successfully developed herbicide-resistant peanut lines by making precise genetic modifications in the *AhALS2* gene [29].

Overall, genome editing techniques, especially the CRISPR system, are proving to be transformative tools in peanut crop production. Advancing the efficiency of transformation and CRISPR technologies remains a priority for future research.

## 6. Conclusions: Future Challenges and Perspectives

The emergence of genome editing technologies has transformed our ability to enhance the genetic traits of crops. This powerful approach enables precise modifications of crop genomes, resulting in improved yields, enhanced disease resistance, and improved nutritional quality. Achieving high precision in genome editing is particularly critical for polyploid species, such as peanuts, which contain multiple sets of chromosomes. The main challenge with these species is accurately targeting specific gene copies while avoiding unintended alterations to other homologous gene copies within their complex genomes.

To achieve the desired genetic modifications without compromising the overall genetic integrity of polyploid crops, the application of advanced techniques and careful planning is essential. While genome editing has significantly accelerated crop improvement efforts, polyploid species, such as peanuts, require an even higher level of precision. Recent studies on the genetic enhancement of peanuts reveal promising opportunities to refine genome editing strategies further. These advances not only contribute to improved food security but also underscore the urgency and importance of ongoing efforts to enhance crop productivity. They highlight the significant impact of our work on the future of agriculture and food security.

**Figure 1:**
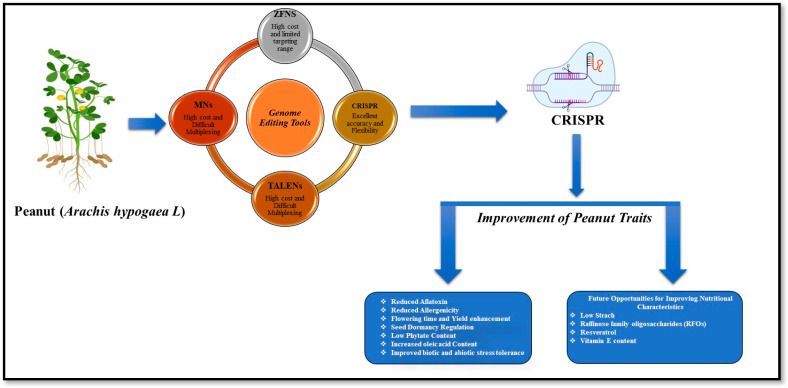
A graphical representation of genome editing aimed at enhancing peanut traits through CRISPR/Cas technology.

Addressing key challenges related to trait improvement in peanuts is critical. These challenges include enhancing tolerance to abiotic stress, managing seed dormancy, reducing allergenicity and fungal contamination in seeds, regulating flowering time, and improving nutritional components such as resistant starch, raffinose family oligosaccharides (RFOs), resveratrol, and vitamin E content (Figure 1). Future collaborative research should focus on optimizing these complex traits for peanut improvement using CRISPR-based genome editing.

This multifaceted research highlights the importance of interdisciplinary collaboration among agronomists, geneticists, and biotechnologists in promoting a collective effort towards a shared goal. It also emphasizes the vital role of refining and harmonizing international regulatory frameworks. This global cooperation and coordination are necessary to facilitate the adoption and trade of genome-edited products. The integration of advanced genome editing tools has the potential to revolutionize peanut breeding and agriculture, helping to overcome current challenges and supporting a more sustainable future for the industry. Ultimately, this will enable the development of novel peanut varieties tailored to meet specific breeding objectives.

## Figures and Tables

**Table 1:** Reports on genome editing tools and genetic transformation methods for improving peanut traits.

Target Gene	Gene Editing Tools/Genetic Transformation Method	Types of Mutation	Transformation Method	Uses	Reference
*ahFAD2A/B*	CRISPR/Cas9	Site-specific In Dels and substitutions disrupting oleate desaturase activity	*Agrobacterium* transformation	Elevated oleic-acid content (≈ 85–90%), reduced linoleic acid, improved oil oxidative stability and nutritional quality	[26]
*Arahy.4E7QKU*	CRISPR/Cas9	Indels	*Agrobacterium* transformation	Improvement of oil quality	[27]
*gfp*	Prime-editing	Site-specific Restoration of mutation	PEG mediated Protoplast	Validation of prime editing	[28]
*AhALS2-A* and *AhALS2-B*	CBE (CRISPR/nCas9)	Base editing	Microprojectile bombardment	Generate herbicide-resistant peanut	[29]
*AhFADH2B*	CRISPR/Cas9	Insertion	*Agrobacterium* and node injection transformation	An increase of more than 80% in oleic acid	[30]
*AhAftr1*	Genetic transformation	-	*Agrobacterium* transformation	Provides resistance to aflatoxin production	[31]
*Ara h 2, Ara h 6, and Ara h 7*	CRISPR/Cas9	Deletion	*Agrobacterium* transformation	Reducing allergens	[32]
*ahFAD2A/ahFAD2B*	CRISPR/Cas9	Transition, Insertion, Transversion	Hairy root Transformation	Functional validation of the *FAD2* gene	[33]
*AhMULE9A*	CRISPR/Cas9	Indels	*Agrobacterium*-mediated pollen tube transformation	Functional validation of *AhMULE9A*	[34]
*AtDREB1A*	Genetic transformation	-	Agrobacterium transformation	Functional validation of *AtDREB1A*	[35]
*AhFAD2*	TALENs	Deletion	Hairy root Transformation	increase in the oleic acid con tent (42.5%–92.5%)	[36]
*AhNFR1* and *AhNFR5*	CRISPR/Cas9	In-Dels	Hairy root Transformation	Functional Validation of nodulation genes	[37]
*RY* and *2S* motif in *AhFAD*	CBE (CRISPR/nCas9)	C to T (47 and 59%), C to G (40 and 26%), and C to A (13 and 15%), G to A	Hairy root Transformation	Demonstration of base editing	[38]

## Data Availability

All information presented in this review comes from previously published sources cited within the text. No new data has been generated or analyzed.
